# Associations Between Peripapillary Retinal Nerve Fiber Layer and Choroidal Thickness With the Development and Progression of Diabetic Retinopathy

**DOI:** 10.1167/iovs.63.2.7

**Published:** 2022-02-02

**Authors:** Xia Gong, Wei Wang, Kun Xiong, Lanhua Wang, Wangting Li, Yuting Li, Meng Yuan, Xiao Guo, Shaochong Zhang, Xiaoling Liang, Hua Liu, Wenyong Huang

**Affiliations:** 1 Zhongshan Ophthalmic Center, State Key Laboratory of Ophthalmology, Sun Yat-Sen University, Guangzhou, People's Republic of China; 2 Jinan University Affiliated Shenzhen Eye Hospital, Guangzhou, People's Republic of China; 3 Department of Ophthalmology, Third Affiliated Hospital of Jinzhou Medical University, Jinzhou, People's Republic of China

**Keywords:** diabetic retinopathy, optical coherence tomography, predictive modeling, prospective cohort, type 2 diabetes

## Abstract

**Purpose:**

To evaluate the role of the peripapillary retinal nerve fiber layer (pRNFL) and peripapillary choroidal thickness (pCT) in the development and progression of diabetic retinopathy (DR).

**Methods:**

This is a cohort study based on the baseline and 2-year follow-up data of the Guangzhou Diabetic Eye Study. Patients with type 2 diabetes mellitus between the ages of 30 and 80 years were recruited from communities in Guangzhou. DR was graded by seven-field fundus photography after dilation of the pupil. pRNFL and pCT were measured via swept-source optical coherence tomography.

**Results:**

A total of 895 patients were included in the study; of these, 748 did not have DR at baseline and 147 had DR at baseline. During the 2-year follow-up, 80 developed DR (10.7%), and 11 experienced DR progression (7.5%). After adjusting for confounding factors, a higher risk of incident DR was strongly associated with a lower average thickness of the pRNFL (risk ratio [RR] per 1 SD, 0.55; 95% confidence interval [CI], 0.42–0.72; *P* < 0.001) and average pCT (RR per 1 SD, 0.49; 95% CI, 0.34–0.70; *P* < 0.001). Adding both metrics to the DR prediction model significantly improved the discriminant ability of the model for incidences of DR (area under the curve increased by 15.38% from 0.673 to 0.777; *P* < 0.001).

**Conclusions:**

Neurodegeneration shown by the thinning of pRNFL and impaired choroidal circulation shown by the thinning of pCT are independently associated with DR onset, and assessing both metrics can improve the risk assessment for DR incidences.

Diabetic retinopathy (DR) is a diabetic complication that affects approximately one-third of diabetic patients^1^ and is the leading cause of avoidable blindness among the working-age population.^2^ Though the pathogenesis of DR remains unclear, growing evidence suggests that retinal neurodegeneration and choroidal impairment may be early events in DR pathogenesis.^3^ Hyperglycemia-induced neurodegeneration, manifested as retinal ganglion cell loss, may precede the detectability of vascular abnormalities in clinical examinations.^4^ Optical coherence tomography (OCT) is a noninvasive technique that captures cross-sectional images, as well as the thickness of different layers of the retina and choroid. The introduction of swept-source OCT has enabled accurate and automatic quantification of peripapillary retinal fiber layer (pRNFL) thickness and peripapillary choroidal thickness (pCT).

A number of cross-sectional studies have reported a decrease in RNFL thickness in diabetic patients, with or without mild DR, compared with normal control subjects.^5^^–^^8^ In addition, it was reported that diabetic patients without any signs of DR had greater RNFL loss in prospective studies, suggesting that retinal neurodegeneration may be an early indicator of DR progression.^9^^,^^10^ However, there is no direct evidence that retinal neurodegeneration is related to the development or progression of microvascular abnormalities. Whether retinal neurodegeneration has any value for predicting DR is still unknown and should be further investigated in a prospective study.^11^

The pRNFL is nourished by choroid vasculature, which provides most of the blood supply the retina needs.^12^ Population-based studies have demonstrated significant associations between pRNFL thickness and pCT in Malaysian adults.^13^ However, the mechanism behind the change in pCT in diabetic patients remains unclear. Several studies have drawn divergent conclusions.^14^^–^^18^ Critically, there is a need to further evaluate the relationship between pCT and DR onset and progression.

Therefore, the objectives of this 2-year follow-up longitudinal study based on a large sample of patients with type 2 diabetic mellitus (T2DM) was to evaluate the associations between the onset and progression of DR and pRNFL thickness and pCT measured via swept-source optical coherence tomography (SS-OCT) at baseline and to determine the predictive value of both pRNFL thickness and pCT as metrics of DR onset and progression.

## Methods

### Subjects

This prospective cohort study was conducted in accordance with the tenets of the Declaration of Helsinki and approved by the Zhongshan Ophthalmic Center Ethics Committee (approval number 2017KYPJ094). Written consent was given by all individuals who participated in the study. This study analyzed the data collected at baseline and follow-up at 2-year interval from October 2017 to December 2020. Ocular treatment–naïve T2DM patients between the ages of 30 and 80 years registered in the healthcare systems of the selected communities were recruited. Patients with any evidence of the following conditions at baseline were excluded: (1) best-corrected visual acuity (BCVA) worse than 20/200; (2) spherical equivalent < −6 diopters (D), astigmatism > +2 D, intraocular pressure > 21 mmHg, and/or axial length (AL) >25.5 mm; (3) vision-threatening diabetic retinopathy (VTDR), defined as severe non-proliferative diabetic retinopathy (NPDR), proliferative diabetic retinopathy (PDR), or diabetic macular edema; (4) any ocular diseases other than DR or refractive errors, such as glaucoma, age-related macular degeneration, and retinal detachment; (5) failure of eye examination due to refracting media opacity and/or poor fixation; (6) severe systemic disease other than DM, such as uncontrolled hypertension, severe cardiovascular and cerebrovascular diseases, malignant tumors, and nephritis; (7) ungradable fundus photographs or OCT images of poor quality; and (8) cognitive disorders or mental illness.

### Retinal Photography and Definition of Outcomes

Standard seven-field fundus photographs (CR-2; Canon, Tokyo, Japan) based on Early Treatment Diabetic Retinopathy Study (ETDRS) criteria were obtained after pupil dilation at baseline and again during the 2-year follow-up. The images were graded by two well-trained graders in accordance with the Diabetic Retinopathy Preferred Practice Pattern guidelines.^19^ DR severity was graded into one of five categories: no DR, mild NPDR, moderate NPDR, severe NPDR, and PDR. Incident DR was defined as newly developed for any incidence of DR during follow-up in participants without DR at baseline. DR progression was defined as progressing at least one level in 2 years.

### SS-OCT Imaging for pRNFL Thickness and pCT

An SS-OCT device (DRI OCT Triton; Topcon, Tokyo, Japan) was used to scan the peripapillary area. SS-OCT uses 1050-nm-wavelength light with a 100-nm-wavelength adjustment range. The longitudinal and transverse resolutions are 8 µm and 10 µm, respectively. A trained technician who was blind to the research protocol examined the participants. The peripapillary region was scanned using a three-dimensional optic disk scanning mode, with a 360°, 3.4-mm-diameter circle that was centered on the optic disc. Scans were centered using an internal fixation. OCT images were automatically segmented into different layers by the built-in software. Then, the same technician carefully inspected and confirmed the segmentation and manually corrected the segmentation when the borderline was misjudged. The pCT was automatically measured and reported as the vertical distance between the retinal pigmented epithelium (the outer portion of the hyperreflective line) and the choroidal–scleral interface (the hyporeflective line). The pRNFL thickness was defined as the vertical distance between the internal limiting membrane and the inner border of the retinal ganglion cell layer. The area was segmented into superior, inferior, temporal, and nasal quadrants automatically. The average pRNFL thickness and pCT in each quadrant were automatically reported. Out-of-focus images and images with motion artifacts or segmentation algorithm failures were classified as unqualified images and excluded from the study.

### Other Systemic and Ocular Variables

All examinations were performed by an experienced nurse in compliance with standard procedures. Systolic blood pressure (SBP) and diastolic blood pressure (DBP) were measured using a blood pressure monitor (Hem-907; Omron, Kyoto, Japan). The mean artery pressure (MAP) was calculated as the sum of one-third of the SBP and two-thirds of the DBP. Body mass index (BMI) was calculated as weight (measured in kilograms) divided by the square of height (measured in meters). Measurements of hemoglobin A1c (HbA1c), total cholesterol (TC), high-density lipoprotein cholesterol (HDL-C), low-density lipoprotein cholesterol (LDL-C), and triglycerides (TG) were made in accordance with standardized procedures at a certified laboratory in China.

Each participant underwent a comprehensive ocular examination that included the BCVA test refraction examination (KR-8800 Auto Refractometer; Topcon), intraocular pressure (IOP) measurement (CT-1 Non-Contact Tonometer; Topcon), slit-lamp examination, and ocular biometric measurements (Lenstar LS900; Haag-Streit, Köniz, Switzerland).

### Statistical Analysis

The data on the right eye or worse eye of each participant were analyzed. The difference in baseline characteristics was evaluated using the independent samples *t*-test for normally distributed data, and the χ^2^ test for qualitative data. Two multivariable logistic regression models were used to evaluate (1) the relationships between pRNFL thickness and pCT, and (2) the incidence and progression of DR. Model 1 included adjustment for age and sex, and Model 2 further adjusted for HbA1c, duration of diabetes, BMI, SBP, DBP, TC, AL, image quality score, and baseline DR severity. The average thickness and thickness in each of the four quadrants of the pRNFL and the pCT were analyzed separately. Evaluation of the additional predictive value of pRNFL thickness and pCT was carried out by calculating the *C* statistic (change in area under the curve [AUC]) upon adding pRNFL thickness and/or pCT to the standard model, which is based on established risk factors—including age, diabetes duration, and HbA1c.^1^ All statistical analyses were performed in Stata/MP 16.0 (StataCorp, College Station, TX, USA), and *P* < 0.05 was considered statistically significant.

## Results

Data on a total of 895 diabetic patients were included in the final analysis, including 748 patients without DR at baseline and 147 patients with DR at baseline. Their average age was 65.0 ± 7.9 years, and the average duration of diabetes was 9.2 ± 7.2 years. After exclusion of VTDR and diabetic macular edema, 28 and 119 participants had mild and moderate NPDR at baseline, respectively. The demographic and clinical characteristics of all participants are presented in Table 1. After 2 years, 80 participants had incident DR (10.7%), and 11 of the DR patients had progression of DR (7.5%). Patients with and without DR development in 2 years had similar characteristics with regard to sex, age, diabetes duration, BMI, MAP, blood lipids, and eye condition. However, Hb1Ac levels were significantly higher in patients who developed DR after 2 years. There was no significant difference in age, diabetes duration, MAP, blood lipids, and eye condition between DR patients with progression and DR patients without progression. Patients with DR progression had higher BMI and Hb1Ac levels and were more likely to be female.

**Table 1.** Baseline Characteristics of Subjects by DR Incidence or Progression

	DR Incidence (*n* = 748)			DR Progression (*n* = 147)		
Characteristics	Without	With	*P*	Without	With	*P*
Subjects, *n*	668	80	—	136	11	—
Female, *n* (%)	390 (63.75)	51 (58.73)	0.388	65 (47.79)	10 (90.91)	**0.001**
Age (y), mean ± SD	64.98 ± 7.96	65.85 ± 8.62	0.361	64.09 ± 8.10	64.04 ± 7.50	0.984
Diabetes duration (y), mean ± SD	8.34 ± 6.72	8.68 ± 7.32	0.678	15.55 ± 5.87	13.47 ± 7.96	0.399
HbA1c (%), mean ± SD	6.57 ± 0.98	7.38 ± 1.56	**<0.001**	9.05 ± 1.72	7.54 ± 1.57	**0.003**
BMI (kg/m^2^), mean ± SD	24.48 ± 3.18	24.81 ± 3.22	0.384	21.16 ± 3.13	23.80 ± 2.68	**0.002**
MAP (mmHg), mean ± SD	177.93 ± 22.17	182.91 ± 23.69	0.060	181.00 ± 22.87	181.29 ± 23.90	0.969
TC (mmol/L), mean ± SD	4.83 ± 1.08	4.77 ± 0.95	0.670	5.01 ± 1.00	4.82 ± 1.22	0.617
Creatinine (µmol/L), mean ± SD	72.33 ± 19.15	77.00 ± 24.95	**0.047**	74.45 ± 11.13	79.13 ± 19.15	0.426
HDL-C (mmol/L), mean ± SD	1.32 ± 0.41	1.32 ± 0.38	0.987	1.52 ± 0.37	1.31 ± 0.37	0.069
LDL-C (mmol/L), mean ± SD	3.05 ± 0.96	3.06 ± 0.79	0.954	3.23 ± 0.99	3.03 ± 0.94	0.497
TG (mmol/L), mean ± SD	2.34 ± 1.61	2.26 ± 2.38	0.690	1.79 ± 0.88	2.49 ± 2.18	0.291
BCVA (logMAR), mean ± SD	0.21 ± 0.12	0.20 ± 0.10	0.303	0.26 ± 0.16	0.23 ± 0.13	0.355
IOP (mmHg), mean ± SD	16.13 ± 2.85	16.76 ± 3.47	0.069	16.19 ± 3.97	16.08 ± 2.99	0.910
CCT (µm), mean ± SD	544.22 ± 30.79	546.13 ± 34.75	0.607	530.36 ± 24.54	546.07 ± 33.52	0.131
AL (mm), mean ± SD	23.50 ± 0.89	23.50 ± 0.89	0.976	22.96 ± 0.46	23.30 ± 0.85	0.192
ACD (mm), mean ± SD	2.49 ± 0.44	2.54 ± 0.55	0.342	2.43 ± 0.19	2.57 ± 0.65	0.468

Bold values indicate statistical significance. CCT, central corneal thickness; ACD, anterior chamber depth.

Table 2 shows the distributions of pRNFL thickness and pCT at baseline against the 2-year incidence and progression of DR. The average pRNFL was significantly thinner in patients with a DR incidence, measuring 111.4 ± 12.9 µm for patients without DR incidence and 103.0 ± 8.8 µm for patients with DR incidence (*P* < 0.001). The difference was significant in the nasal, inferior, and superior quadrants (*P* < 0.05). The average pCT was significantly thinner in patients with DR incidence, measuring 114.4 ± 52.6 µm for patients without DR incidence and 91.1 ± 33.4 µm for patients with DR incidence (*P* < 0.001). In each peripapillary quadrant, there was significant thinning of the pCT in patients with DR incidence (*P* < 0.05). There was no statistical difference in the pRNFL or pCT of DR patients with progression and DR patients without DR progression after 2 years. Because the measure of the two metrics could be affected by a magnification effect,^20^ subgroup analyses were performed after participants were divided into two group according to the median of their axial length (23.43 mm). Supplementary Table S1 shows that the thinning of average pRNFL thickness and pCT was still significantly correlated with a higher risk of 2-year DR incidence in each subgroup (all, *P* < 0.05).

**Table 2.** Baseline SS-OCT Metrics in Peripapillary Region by 2-Year Incidence or Progression of DR

	DR Incidence, mean ± SD			DR Progression, mean ± SD		
Parameters	Without	With	*P*	Without	With	*P*
pRNFL (µm)						
Nasal quadrant	83.7 ± 18.8	77.2 ± 14.5	**0.003**	81.2 ± 19.0	74.6 ± 13.4	0.335
Temporal quadrant	82.5 ± 17.8	81.4 ± 16.8	0.587	83.0 ± 18.1	83.9 ± 19.1	0.898
Superior quadrant	134.0 ± 21.1	123.9 ± 16.9	**<0.001**	131.8 ± 19.2	141.3 ± 21.7	0.185
Inferior quadrant	145.3 ± 24.1	129.2 ± 15.5	**<0.001**	142.7 ± 24.3	146.5 ± 27.9	0.669
Average	111.4 ± 12.9	103.0 ± 8.8	**<0.001**	109.7 ± 13.7	111.6 ± 13.8	0.701
pCT (µm)						
Nasal quadrant	118.3 ± 60.6	94.1 ± 42.9	**<0.001**	130.1 ± 68.8	121.2 ± 35.6	0.702
Temporal quadrant	120.8 ± 61.4	95.0 ± 38.5	**<0.001**	128.2 ± 69.6	114.1 ± 38.4	0.550
Superior quadrant	125.8 ± 58.3	99.1 ± 36.8	**<0.001**	131.4 ± 64.0	127.0 ± 42.0	0.841
Inferior quadrant	92.5 ± 46.1	75.6 ± 32.6	**<0.001**	96.1 ± 51.2	88.2 ± 31.3	0.651
Average	114.4 ± 52.6	91.1 ± 33.4	**<0.001**	121.5 ± 60.1	112.4 ± 34.6	0.656

Bold values indicate statistical significance.

Table 3 outlines the associations between baseline SS-OCT metrics in the peripapillary region and the risk of 2-year incidence of DR. After adjusting for age, sex, HbA1c, duration of diabetes, BMI, SBP, DBP, TC, AL, image quality score, and baseline DR severity, reduced pRNFL thickness and pCT were negatively associated with a higher risk of incident DR (average pRNFL: risk ratio [RR], 0.55; 95% confidence interval [CI], 0.42–0.72; average pCT: RR, 0.49; 95% CI, 0.34–0.70). Subregion analysis yielded consistent results (*P* < 0.05) except for pRNFL in the temporal quadrant (*P* = 0.587). However, there was no statistically significant relationship between the baseline values of pRNFL thickness and pCT in the peripapillary region and the risk of DR progression within 2 years (Table 4).

**Table 3.** Baseline SS-OCT Metrics in Peripapillary Region and Risk of 2-Year Incidence of DR

	Model 1^*^		Model 2^†^	
	RR (95% CI)	*P*	RR (95% CI)	*P*
pRNFL (per 1-SD increase)				
Nasal quadrant	0.66 (0.51–0.87)	**0.003**	0.72 (0.55–0.95)	**0.022**
Temporal quadrant	0.94 (0.73–1.19)	0.587	0.99 (0.78–1.27)	0.965
Superior quadrant	0.62 (0.50–0.79)	**<0.001**	0.68 (0.53–0.86)	**0.002**
Inferior quadrant	0.51 (0.40–0.65)	**<0.001**	0.51 (0.39–0.66)	**<0.001**
Average	0.51 (0.40–0.66)	**<0.001**	0.55 (0.42–0.72)	**<0.001**
pCT (per 1-SD increase)				
Nasal quadrant	0.60 (0.44–0.80)	**0.001**	0.54 (0.39–0.76)	**<0.001**
Temporal quadrant	0.57 (0.42–0.77)	**<0.001**	0.55 (0.39–0.78)	**0.001**
Superior quadrant	0.54 (0.40–0.74)	**<0.001**	0.51 (0.36–0.72)	**<0.001**
Inferior quadrant	0.61 (0.45–0.83)	**0.002**	0.54 (0.37–0.78)	**0.001**
Average	0.55 (0.40–0.75)	**<0.001**	0.49 (0.34–0.70)	**<0.001**

Bold indicates statistical significance.

* Adjusted for age and sex.

† Further adjusted for HbA1c, duration of diabetes, BMI, SBP, DBP, TC, AL, and image quality score.

**Table 4.** Baseline SS-OCT Metrics in Peripapillary Region and Risk of 2-Year DR Progression

	Model 1^*^		Model 2^†^	
	RR (95% CI)	*P*	RR (95% CI)	*P*
pRNFL (per 1-SD increase)				
Nasal quadrant	0.65 (0.28–1.52)	0.321	0.54 (0.22–1.33)	0.179
Temporal quadrant	1.05 (0.52–2.11)	0.897	1.05 (0.53–2.09)	0.887
Superior quadrant	1.77 (0.77–4.08)	0.182	2.17 (0.89–5.28)	0.087
Inferior quadrant	1.17 (0.57–2.38)	0.665	1.23 (0.59–2.57)	0.577
Average	1.14 (0.59–2.18)	0.697	1.17 (0.60–2.27)	0.648
pCT (per 1-SD increase)				
Nasal quadrant	0.88 (0.46–1.69)	0.699	0.92 (0.44–1.93)	0.819
Temporal quadrant	0.81 (0.41–1.61)	0.547	0.70 (0.28–1.72)	0.433
Superior quadrant	0.94 (0.49–1.78)	0.839	0.95 (0.47–1.93)	0.891
Inferior quadrant	0.85 (0.42–1.70)	0.649	0.85 (0.35–2.04)	0.712
Average	0.86 (0.45–1.66)	0.653	0.84 (0.38–1.87)	0.675

* Adjusted for age and sex.

† Further adjusted for HbA1c, duration of diabetes, BMI, SBP, DBP, TC, AL, image quality score, and baseline DR severity.

We further evaluated whether pRNFL thickness and/or pCT could add predictive value to the standard model, which is based on traditional risk factors for DR (i.e., age, diabetes duration, and HbA1c). Table 5 presents the AUC analysis for predictive models for the 2-year incidence of DR. The AUC for the standard predictive model was 0.673 (CI, 0.605–0.742). We found significant improvements in AUC after pRNFL thickness and pCT were added to the standard predictive model separately (for pRNFL, the AUC increased by 11.80% from 0.673 to 0.753, *P* < 0.001; for pCT, AUC increased by 7.80% from 0.673 to 0.726, *P* = 0.014). The addition of both pRNFL thickness and pCT significantly improved AUC for any incidence of DR by 15.38% from 0.673 to 0.777 (*P* < 0.001). The receiver operating characteristic curves for the 2-year incidence of DR are presented in the Figure.

**Table 5.** AUC Analysis for Predictive Models of 2-Year DR Incidence

	OCT Metrics Added to Predictive Model			
OCT Metrics	Without (95% CI)	With (95% CI)	AUC Increase (%)	*P*
Average pRNFL	0.673 (0.605–0.742)	0.753 (0.700–0.806)	11.80 (8.68–15.66)	**<0.001**
Average pCT	0.673 (0.605–0.742)	0.726 (0.663–0.789)	7.80% (6.40–9.52)	**0.014**
Average pRNFL and pCT	0.673 (0.605–0.742)	0.777 (0.725–0.829)	15.38 (11.78–19.79)	**<0.001**

Bold values indicate statistical significance. Standard model is based solely on established risk factors (age, diabetes duration, and HbA1c).

**Figure.**
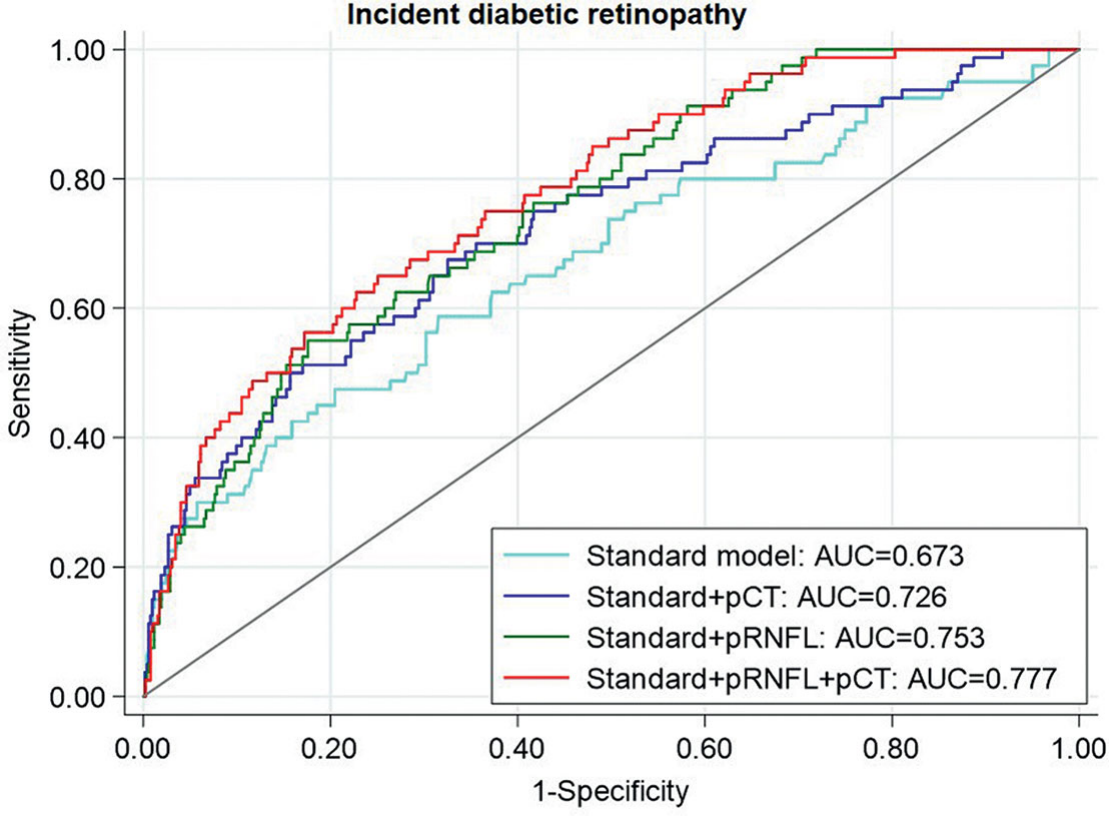
Receiver operating characteristic curves of predictive models of 2-year incidence of diabetic retinopathy. The *bright blue curve*, *dark blue curve*, *green curves*, and *red curve* represent the receiver operating characteristic curves for the standard model, standard model plus pCT, standard model plus pRNFL thickness, and the standard model plus pRNFL thickness plus pCT, respectively.

## Discussion

To the best of our knowledge, this study is the first large-sample, prospective study to evaluate the relationships between pRNFL thickness and pCT and the incidence and progression of DR in ocular treatment-naïve patients with T2DM. The main findings are as follows: (1) The average pRNFL thickness and pCT were significantly thinner in patients who had an incidence of DR within the 2-year study period. (2) The average pRNFL thickness and pCT were independently correlated with DR incidences after adjustment for other confounding factors. (3) Considering pRNFL thickness and pCT improved the ability of the standard model to predict the incidences of DR. Our findings provide an important basis for research on neurodegeneration and the development of DR and demonstrate the potential of OCT to detect early changes before the development of clinically evident DR.

An increasing body of evidence suggests that retinal neurodegeneration plays a significant role in the pathogenesis of DR. Cross-sectional studies using OCT report a decrease in RNFL thickness in diabetic patients with or without mild DR when compared with normal controls.^5^^–^^9^ However, RNFL thickness for different levels of severity of DR shows conflicting results. Marques et al.^21^ reported no difference and Li et al.^22^ reported an increasing trend in RNFL thickness with increasing DR severity. Furthermore, Lim et al.^9^ found a greater loss of pRNFL in 103 patients with T2DM than in 63 healthy individuals over the course of 3 years, with the decline being even more pronounced in patients with mild DR. Lee et al.^10^ also found a greater decrease over 3 years in 85 diabetic patients without DR when compared with 55 normal participants. Our study advances the findings of the aforementioned studies and provides strong evidence that a decrease in pRNFL thickness is related to a higher risk of an incidence of DR. This finding can guide further research into the role of neurodegeneration in the development of DR.

The reduction in temporal pRNFL thickness in diabetic eyes has been reported by many researchers.^7^^,^^9^ However, our results indicate no significant difference in temporal pRNFL between patients without DR incidence and patients with DR incidence. This could because the temporal quadrant has the thinnest pRNFL compared with other quadrants, so the change may be more difficult to observe until later stages of neurodegeneration.^23^ Temporal RNFL is also found to be the most conserved quadrants in glaucoma, another neurodegenerative eye disease.^23^^,^^24^

Current research on choroidal thickness in diabetic patients is still controversial. In cross-sectional studies,^14^^–^^18^ choroidal thickness in diabetic patients was reported to be thinner, to be thicker, or to show no statistical difference compared with normal control subjects. Our previous study found that choroidal thickness increased during the early stages of DR and then decreased with further DR progression,^14^ suggesting that the change in choroidal thickness may be different for various levels of DR severity. The divergent findings of this study may be attributable to differences in the consideration of confounding factors, the use of OCT instruments, and insufficient sample sizes for subgroup analysis of patients at different stages of DR. Furthermore, the relationship between choroidal thickness and incidences of DR or DR progression is even less clear due to a dearth of longitudinal evidence. Our findings do confirm, however, that a thinner pCT is related to a higher risk of developing DR.

With the participants in our study, there were statistically significant changes in the AUC when pRNFL thickness, pCT, or both metrics were added to the predictive model for DR incidences. To the best of our knowledge, the predictive value of pRNFL thickness and pCT for DR has not been investigated in any previous studies. The pRNFL thickness and pCT should be considered as components in the development of more accurate DR predictive models as the non-invasive OCT becomes more available in the future. This will reduce the cost-effectiveness ratio of DR screening by prolonging the screening interval for patients at low risk of developing DR.

No statistical significance was found between baseline values of pRNFL thickness and pCT and the risk of DR progression within 2 years. There are two possible reasons for this. One reason could be the comparatively small sample size of DR patients in our study. Participants with diabetes were voluntarily recruited from communities, most of whom had no symptoms of DR. Moreover, we excluded patients with VTDR at baseline because such patients need intense treatment rather than annual follow-up. We then further excluded patients with DR but who had received laser or surgery treatment at baseline. Another reason could be the low progression rate. Only 11 in 147 DR patients had progression after 2 years. Thus, the relationship among pCT, pRNFL, and DR progression should be evaluated in larger sample sizes with longer follow-up.

The neurovascular unit (NVU) in the retina, which is defined as a coupling and interdependency of neurons, is essential for maintaining the structure and function of the blood–retinal barrier. Impairment of the NVU induced by hyperglycemia may be an early event in the pathogenesis of DR. The combined impact of NVU impairment, a dysfunctional basement membrane, pericyte loss, and endothelial damage may cause disruption of the blood–retinal barrier and eventually result in clinically evident DR.^3^ As the choroid is closely related to retinal structural and functional changes,^12^^,^^13^ the relationship between the choroid and DR, a chronic retinal disease, has become a hot research topic. Because a thinner peripapillary choroid has been found to be independently associated with a thinner RNFL,^13^ it is supposed that choroid changes may play a role in retinal neurodegeneration; however, the mechanism remains unclear. Because the choroid supplies most of the blood supply that the retina needs^12^ and choroid thinning indicates a decrease in blood flow, we hypothesized that choroid thinning leads to hypoxia in retinal tissue, which may further lead to retinal neurodegeneration. Further experiments are necessary to confirm this.

As mentioned above, previous observational studies in humans have not directly identified the causes and effects for neurodegeneration, choroid structural changes, and incidence of DR; however, our study demonstrates a direct link between neurodegeneration, choroidal changes, and DR incident. Combined with previous experimental evidence, it is confirmed that neurodegeneration plays an important part in the development of DR. Therefore, studying the underlying mechanisms that lead to NVU destruction and later neurodegeneration is critical for the development of novel therapeutic strategies.^3^^,^^11^

The strength of our study is that, to our knowledge, it is the first prospective study to evaluate the relationship among pRNFL thickness, pCT, and DR incidence and progression in patients with T2DM, as well as to illuminate the potential predictive value of both metrics in predicting DR incidence. Another strength of this study is the inclusion of a relatively large sample of T2DM patients with no history of eye treatment, with all participants recruited from homogeneous communities. Third, we applied the following measures to enhance the credibility of our study: strict compliance with implementation procedures, use of the golden standard of DR grading (ETDRS seven-field fundus photography), and adjustment for a variety of potential confounding factors.

There are also limitations. First, this is not a population-based study, and, as such, selection bias may impact the results; however, our findings still provide some reference value because the results are based on a large and homogeneous sample. Second, this study included only Chinese patients with T2DM; however, patients with a different type of diabetes mellitus or a different subgroup of T2DM,^25^ or patients of a different race,^26^ may have different characteristics—including DR incidence. Thus, the generalization of our findings to other patients should be considered with caution; however, because diabetic patients of different races have similar risk factors for DR,^26^ we believe that the proven relationships apply to other populations, as well. Third, a 2-year follow-up may be too short for slow-developing DR; however, we found the 2-year incidence and progression rates to be consistent with those of previous report,^1^ indicating the reliability and robustness of the findings. Fourth, the relatively small sample size of DR patients at baseline and the short-term follow-up may be the reason why we did not find any significant association between pRNFL thickness or pCT and DR progression. Further study with a large sample is required to confirm or refute our results. Fifth, participants were not scanned at the same time during the day, but it has been suggested that pCT could change with circadian rhythm.^27^^–^^29^ We took this issue into account during the design of the study, as the scanning times for the participants were not deliberately arranged based on their different conditions but instead were randomly arranged based on the time of their checkpoint. ANOVA analysis showed no difference in pCT among groups who had different scan times (*P* = 0.637). Overall, we believe that the exam time has little effect on the results.

In conclusion, the findings of this prospective longitudinal observational study prove that pRNFL thickness and pCT are significantly correlated with DR incidence. Furthermore, both pRNFL thickness and pCT have predictive value for predicting DR incidence. These results illuminate a strong connection between neurodegeneration and DR incidence and suggest that pRNFL thickness and pCT are potential components of a future predictive model. However, our findings should be verified for other populations and longitudinal cohorts with a longer follow-up period, in addition to further exploration of the underlying mechanisms of neurodegeneration in the development of DR.

## Supplementary Material

Supplement 1
