# Supplementary material for: Associations Between Peripapillary Retinal Nerve Fiber Layer and Choroidal Thickness With the Development and Progression of Diabetic Retinopathy
Source: Invest Ophthalmol Vis Sci. 2022 Feb 2;63(2):7. doi: 10.1167/iovs.63.2.7 (PMC8819282; doi:10.1167/iovs.63.2.7)
Supplement: Supplement 1 [file iovs-63-2-7_s001.pdf]

Supplementary Table 1. Baseline Swept-source OCT metrics in peripapillary region after median stratification of the axial length and risk of 2-year incident diabetic retinopathy.

	**Model 1***		**Model 2†**	
	**RR (95%CI)**	**P-value**	**RR (95%CI)**	**P-value**
**Average pRNFL**				
Axial length < 23.43 mm	0.42 (0.28, 0.63)	**< 0.001**	0.61 (0.42, 0.87)	**0.007**
Axial length ≥ 23.43 mm	0.52 (0.33, 0.81)	**0.004**	0.59 (0.40, 0.87)	**0.008**
All eyes	0.51 (0.40, 0.66)	**<0.001**	0.55 (0.42, 0.72)	**<0.001**
**Average pCT**				
Axial length < 23.43 mm	0.43 (0.24, 0.74)	**0.003**	0.56 (0.34, 0.93)	**0.025**
Axial length ≥ 23.43 mm	0.40 (0.22, 0.71)	**<0.001**	0.57 (0.35, 0.94)	**0.029**
All eyes	0.55 (0.40, 0.75)	**<0.001**	0.49 (0.34, 0.70)	**<0.001**

Abbreviations: RR=risk ratio; 95%CI=95% confidential interval; pRNFL=peripapillary retinal nerve fibre layer thickness; pCT=peripapillary choroidal thickness.

Bold indicates statistical significance.

*Adjusted for age and sex.

†Further adjusted for HbA1c, duration of diabetes, BMI, SBP, DBP, total cholesterol, axial length and image quality score.
